# Impact of extent of parietal peritonectomy on oncological outcome after cytoreductive surgery and HIPEC

**DOI:** 10.1515/pp-2019-0015

**Published:** 2019-11-06

**Authors:** S.P. Somashekhar, K.R. Ashwin, Ramya Yethadka, Shabber S. Zaveri, Vijay K Ahuja, Amit Rauthan, Kumar C. Rohit

**Affiliations:** Department of Surgical Oncology, Manipal Comprehensive Cancer Centre, Manipal Hospital, Bengaluru India; Department of Medical Oncology, Manipal Comprehensive Cancer Center, Manipal Hospital, Bangalore, Karnataka, India

**Keywords:** cytoreductive surgery, hyperthermic intraperitoneal chemotherapy (HIPEC), peritoneal surface malignancy, peritonectomy

## Abstract

**Background:**

In peritoneal surface malignancy (PSM), in spite of optimal cytoreductive surgery (CRS), majority of recurrences that occur are intraperitoneal. In patients with PSM, studies employing fluorescent imaging and microscopic examination have shown normal looking peritoneum may harbor active disease. This study was done to assess the recurrence pattern, oncological outcomes, and morbidity and mortality of the extent of peritonectomy in patients who underwent total parietal peritonectomy (TPP) or involved field peritonectomy (IFP) as a part of the procedure during CRS and hyperthermic intraperitoneal chemotherapy (HIPEC).

**Methods:**

This was a retrospective analysis of prospectively collected data, from February 2013 to December 2017. A total of 163 patients with PSM underwent TPP or IFP with CRS plus HIPEC. Their oncological outcomes, recurrence pattern, postoperative morbidity and mortality were analyzed.

**Results:**

Of the 163 cases, the primary organs of origin were ovary, colorectal, appendicular pseudomyxoma, stomach, mesothelioma and others (67.4%, 16.5%, 6.1%, 4.9%, 2% and 2%), respectively. TPP was performed in 70 patients and IFP in 93 patients. TPP group had higher mean PCI (16 vs. 14), longer duration of surgery (11 vs. 9 h), and more blood loss (1,243 vs. 675 mL). Overall G3–G4 morbidity was comparable in both groups (42.8% vs. 33.3%) as was mortality (5.7% vs. 4.4%). Kaplan–Meier analysis showed that with a median follow-up of 45 months, TPP group had a recurrence-free survival (RFS) of 26 months and overall survival (OS) was yet to be achieved, whereas the IFP group had a RFS and OS of 21 and 43 months, respectively.

**Conclusions:**

Performing TPP reduces the chance of missing the microscopic disease, therefore can minimize local recurrence, and better oncological outcomes. TPP can be performed with acceptable morbidity and mortality, at the cost of increased duration of surgery and higher blood loss.

## Introduction

Peritoneal surface malignancies (PSM) occur by the spread and implantation of tumor cells throughout the peritoneal cavity [1]. Cytoreductive surgery (CRS) with or without adjuvant systemic chemotherapy has been the standard treatment for PSM. In spite of this, PSM is known to have dismal prognosis with majority of recurrences being intraperitoneal.

Optimal CRS is the cornerstone in the management of PSM with curative intent. It comprises of complete removal of macroscopic disease, so as to achieve a minimal residual disease of less than 2.5 mm^2^. Completeness of cytoreduction score (CC Score) has been found to be an important predictor of long term outcome after CRS in PSM of colorectal origin [2], ovary [3] and pseudomyxoma peritonei (PMP) [4].

CRS involves peritonectomy procedures with or without en-bloc resection of the involved viscera. Peritonectomy is an essential component in management of PSM [1]. Parietal peritoneum constitutes only about 30% of total peritoneum, while the rest is by visceral peritoneum. Thus, the complete visceral peritonectomy [5] might need visceral resections majority of the times (except in case of mesenteric peritonectomy). However, there is no consensus regarding the extent of the peritonectomy procedure to be done. Standard of care today is the removal of the involved part of peritoneum (involved field peritoneum – IFP) and viscera. Immunofluorescence studies [6, 7] and histopathological examination done after IFP have shown disease in residual peritoneum not suspected on gross examination. Disease burden may be underestimated in implants size <5mm, after neoadjuvant chemotherapy (NACT) or after previous surgery. This stresses the need for total removal of parietal peritoneum (total parietal peritonectomy – TPP) so as to achieve complete cytoreduction, while questioning the role of IFP. The role of TPP has been investigated in malignant peritoneal mesothelioma [8] and PSM of ovarian origin [9].

Use of perioperative intraperitoneal chemotherapy has been postulated to improve control of peritoneal disease, due to the better loco regional tissue penetration of chemotherapy drugs with reduced systemic toxicity [10]. Normothermic intraperitoneal (IP) port based adjuvant chemotherapy or early postoperative intraperitoneal chemotherapy (EPIC) are the intraperitoneal chemotherapy forms used in various treatment protocols. Hyperthermic intraperitoneal chemotherapy (HIPEC), a form of intraperitoneal chemotherapy, has emerged recently as a novel option for the treatment of patients with PSM. It has the added advantage of single shot delivery done at the time of surgery, homogenous distribution and synergistic effect of heat but additional morbidity [11]. Randomized controlled trials [12, 13, 14] have shown improved outcomes with CRS plus HIPEC in terms of overall survival (OS) and recurrence-free survival (RFS) with acceptable morbidity and mortality rates in case of epithelial ovarian carcinoma and colorectal carcinoma. However, there still exists wide skepticism about the benefits of CRS plus HIPEC, as well as concerns about its complications.

We postulated that optimal CRS along with TPP may be advantageous for better local control thus improving the oncologic outcomes. Therefore, the present retrospective study was done to assess the oncological outcomes, RFS and OS, recurrence pattern, morbidity and mortality of the extent of parietal peritonectomy (IFP or TPP) in Indian patients with PSM undergoing CRS with HIPEC.

## Materials and methods

### Methodology

This was a retrospective study of prospectively collected data, done at Manipal Comprehensive Cancer Center, Manipal Hospital, from February 2013 to December 2017. Patients diagnosed with PSM from various primary cancers underwent CRS and HIPEC. The patient cohort included cases undergoing upfront surgery, interval cytoreduction post NACT and those undergoing surgery for recurrent disease. Patients with PSM, without distant metastasis, Eastern Cooperative Oncology Group (ECOG) performance status <2 and preoperative serum albumin >3 g% were included in the study. Patients with known allergy to intraperitoneally administered chemotherapeutic agents, patients with poor hepatic, respiratory, cardiac, kidney (creatinine clearance <60 mL/min according to the Cockfort formula) or bone marrow function (platelet count <150,000/μL, absolute neutrophil count <1,500/mm^3^) were excluded. Informed consent was obtained from all patients. Ethics committee (EC) approval and Institutional Review Board (IRB) approval was obtained. All patients were treated by a team of two surgeons, anesthesiologist, intensivist and medical oncologist having expertise in PSM.

**Figure 1:**
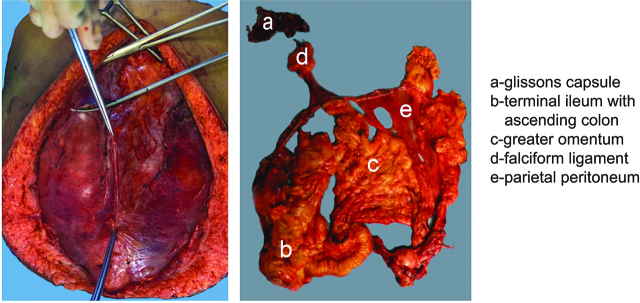
Demonstration of total parietal peritonectomy with multivisceral resection for peritoneal surface malignancy.

Laparotomy was done by midline vertical incision. Peritoneal carcinomatosis index (PCI) [15] was calculated. Optimal CRS with or without visceral resection was done. Patients who underwent TPP had en-bloc stripping and resection of anterior parietal peritoneum, pelvic peritoneum, bilateral diaphragmatic peritoneum, supracolic greater omentectomy with lesser omentectomy (Figure 1(A) and 1(B)). IFP group had stripping and resection of involved peritoneum with visible disease. Organ resections were done whenever involved by the tumor deposits. Multivisceral resection was defined as>2 organs or parts resected. Completeness of cytoreduction (CC) score was documented and HIPEC was done in patients with CC scores 0 and 1. HIPEC was performed by colesium technique with hyperthermia machine as per the institutional protocol [16]. Patients were observed in a high dependency unit (HDU) for the first 24–48 h.

(A) Intraoperative demonstration of total anterior parietal peritonectomy. (B) Specimen of total parietal peritonectomy with visceral resection.

Baseline patient demographics and perioperative details such as PCI, duration of surgery, average blood loss and hospital stay were recorded prospectively. Postoperative morbidity was graded using CTCAE-NCI V 4.03 and Clavien-Dindo classification [17]. Patients were followed-up with clinical assessment, tumor markers and radiological monitoring. Early postoperative morbidity (within 30-postop days), mortality, pattern of recurrence, RFS and OS were calculated. RFS was defined in months as time from day of surgery to first recurrence or last follow-up whichever was the earliest and OS as time from day of surgery to death or last follow-up.

### Statistical analysis

The descriptive statistics including mean and standard deviation (SD) for continuous data and frequencies and percentages for categorical data were calculated. The correlations of the variables with the parameters were calculated by Student t test for continuous variables and Chi-square test for non-continuous variables. Statistical significance was defined as p<0.05 with 95% confidence interval (CI). Survival was calculated by Kaplan–Meier survival analysis. The data were recorded according to the institutional rules, including electronic archiving and video recording of the procedures. Statistical analysis was performed with SPSS-22 (SPSS Inc., Chicago, IL, USA).

## Results

From February 2013 to December 2017, 163 patients diagnosed with PSM from various primary cancers underwent CRS with HIPEC. Of the 163 cases, the primary organ of origin were ovary (67.4%), colorectal (16.5%), appendicular pseudomyxoma (6.1%), stomach (4.9%) and mesothelioma (2.4%). Prior surgical score was 0, 1, 2 and 3 in 101, 18, 38 and 6 patients, respectively. Upfront (n=38), interval (n=76) and recurrent (n=49) cytoreduction were performed based on the timeline at presentation. Patients were grouped into IFP (n=93) and TPP (n=70) groups, based upon the extent of peritonectomy done. Patients’ baseline characteristics and demographics were comparable between both groups.

Patients in TPP group had higher PCI (16 vs. 14; p=0.45), longer mean duration of surgery (11 vs. 9 h; p<0.05), higher intraoperative blood loss (1,243 vs. 675 mL; p<0.05) and increased duration of hospital stay (16 vs. 12 days; p<0.05) when compared to IFP group. Table 1 shows relevant patient demographics, disease characteristics, and perioperative outcomes.

**Table 1:** Baseline characteristics and perioperative outcomes.

Characteristics	Involved field peritonectomy (IFP) (n=93)	Total parietal peritonectomy (TPP) (n=70)	p
Age, years, mean ± SD	54.1 ± 10.74	53.5 ± 9.78	0.737
Gender (male:female), n (%)	79 (84.95):14 (15.05)	49 (70):21 (30)	
ECOG, n (%)			
0	83 (89.25)	61 (87.14)	
1	10 (10.75)	9 (12.86)	
Hemoglobin g%, mean ± SD	10.8 ± 1.44	11.2 ± 1.63	0.082
Albumin g%, mean ± SD	3.9 ± 0.46	3.9 ± 0.48	0.658
Site, n (%)			
Ovary	70 (75.27)	40 (57.14)	
Colorectal	14 (15.05)	13 (18.57)	
Gastric	7 (7.53)	1 (1.43)	
Pseudomyxoma peritonei	0	10 (14.29)	
Mesothelioma	0	4 (5.71)	
Others (endometrial, small bowel adenocarcinoma)	2 (2.15)	2 (2.86)	
Co-morbidity, n (%)	54 (58.06)	39 (55.71)	0.34
Prior surgical score (PSS)			
PSS 0 101 PSS 1 18 PSS 2 38 PSS 3 6	59 (63%) 10 (10.7%) 22 (23.6%) 4 (4.3%)	42 (60%) 8 (11%) 16 (22%) 2 (2.8%)	
Primary disease, n (%)			
Upfront surgery	9 (9.68)	10 (14.29)	0.582
Interval surgery	52 (55.91)	41 (58.57)	0.579
Recurrentdisease, n (%)	32 (34.41)	19 (27.14)	0.309
CC score, n (%)			
0	82 (88.17)	63 (90)	
1	11 (11.83)	7 (10)	
Intra-operative variables,mean ± SD			
PCI score	14.0 ± 6.34	16.0 ± 9.83	0.45
Duration ofsurgery (h)	9.2 ± 1.93	11.2 ± 2.25	<0.05
Blood loss (mL)	675.3 ± 402.1	1,243.6 ± 707.4	<0.05
ICU stay (days)	2.5 ± 3.22	4.3 ± 5.55	<0.05
Gastrointestinal recovery	5.5 ± 2.2	7.1 ± 3.21	<0.05
Hospital stay (days)	12.5 ± 5.52	16.1 ± 9.20	<0.05

ECOG, Eastern Cooperative Oncology Group; n, number; IFP, involved field paritonectomy; TPP, Total parietal paritonectomy; PCI, percutaneous coronary interventions; SD, standard deviation; CC, complete cytoreduction; ICU, intensive care unit.

TPP group had increased diaphragmatic resections (50% vs. 33.3%; p=0.024), bowel resections (65.7% vs. 50.5%; p=0.037), bowel anastomosis (61.4% vs. 47.3%; p=0.018) and multivisceral resections (32.9% vs. 9.7%; p<0.001) when compared to IFP group (Table 2).

**Table 2:** Visceral resections.

Procedures	Involved field peritonectomy (IFP) (n=93)	Total parietal peritonectomy (TPP) (n=70)	p
Diaphragm resection	31 (33.3%)	35 (50%)	0.024
Bowel resection	47 (50.5%)	46 (65.7%)	0.037
Anastomosis	44 (47.3%)	43 (61.4%)	0.018
Stoma	9 (9.7%)	9 (12.9%)	0.346
Multivisceral resection	9 (9.7%)	23 (32.9%)	<0.001
Mesenteric stripping	3 (3.2%)	4 (5.7%)	0.45
Gastric resection	2 (2.1%)	6 (8.5%)	0.34
Glisson’s capsulectomy	10 (10.7%)	15 (21.4%)	0.25
Bladder resection	2 (2.1%)	8 (11.4%)	0.08

Postoperative morbidity in terms of grades 3–4 electrolyte imbalance, hematological toxicity, renal morbidity and cardiac toxicities were comparable in both groups. TPP group had increased intra-pleural and intra-abdominal collections which needed intervention in the form of therapeutic aspiration. Overall grades 3–4 postoperative morbidity was comparable in IFP and TPP groups (33.3% vs. 42.8%; p=0.21). The morbidity outcomes are shown in Table 3. The 30-day mortality was 4 (4.4%) and 4 (5.7%) in IFP and TPP groups (p=0.15), respectively.

**Table 3:** Morbidity outcomes.

Morbidity, n (%)	Involved field peritonectomy (IFP) (n=93)	Total parietal peritonectomy (TPP) (n=70)	p
Electrolyte imbalance Grades 1–2	70 (75.3%)	49 (71%)	0.33
Grades 3–4	12 (12.9%)	10 (14.5%)	0.47
Hematological abnormality Grades 1–2	60 (64.5%)	51 (72.9%)	0.16
Grades 3–4	14 (15.1%)	14 (20%)	0.26
Acute kidney Injury Grades 1–2	26 (28%)	18 (25.7%)	0.44
Grades 3–4	2 (2.2%)	8 (11.4%)	0.01
Pulmonary complications Grades 1–2	17 (18.3%)	19 (27.1%)	0.12
Grades 3–4	9 (9.7%)	19 (27.1%)	0.01
Cardiac complication Grades 1–2	4 (4.3%)	2 (2.9%)	0.48
Grades 3–4	2 (2.3%)	5 (7.1%)	0.12
Surgical morbidity Grade 3 intra-abdominal collection	15 (16.1%)	29 (41.4%)	0.01
Intestinal perforation	2 (2.2%)	4 (5.7%)	0.21
Relaparotomy	7 (7.5%)	8 (11.4%)	0.27
G3–G5 morbidity overall	31 (33.3%)	30 (42.8%)	0.21
G3 surgical morbidity	9 (9.7%)	9 (12.9%)	0.34
Recurrence pattern, %			
Overall	53.7%	40%	–
Peritoneal recurrence	60%	35.7%	–

Twenty-one of 70 patients (30%) in the TPP group had microscopic tumor deposits involving the peritoneum, detected during pathological analysis in areas where no visually evident tumor was detected by the surgeon.

Overall recurrence rate in IFP group was 53.7%. The sites of recurrence was peritoneal in 60%, lymph nodes in 20%, visceral in 20% and 12% had extra abdominal recurrences. TPP group had an overall recurrence rate of 40% most of which were visceral (42.8%) followed by retroperitoneal lymph nodal (39%), peritoneal (35.7%) and extra-abdominal (18%).

**Figure 2:**
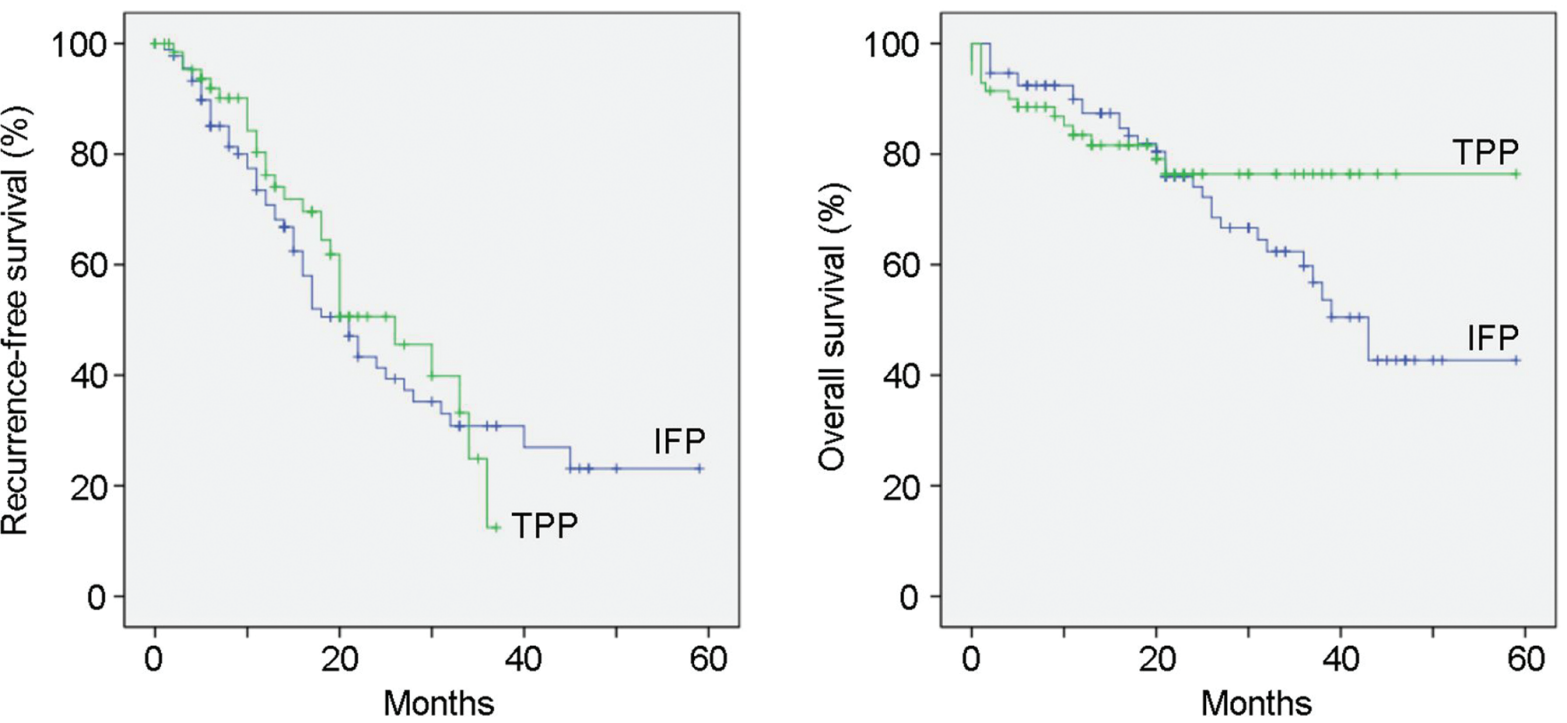
Kaplan–Meier survival curves of total parietal peritonectomy (TPP) and involved field peritonectomy (IFP).

The median duration of follow-up was 45 months. Kaplan–Meier curve for RFS and OS are detailed in Figure 2. The median overall RFS was 21 months. Median RFS in IFP and TPP groups was 21 and 26 months, respectively. Median OS was 43 months in IFP group and was yet to be achieved in TPP group. Three-year OS was 60% in IFP group vs. 80% in TPP group, and 4-year OS was 42% in IFP group vs. 80% in TPP group.

(A) Recurrence-free survival (RFS) of total parietal peritonectomy (TPP) and involved field peritonectomy (IFP). (B) Overall survival (OS) of total parietal peritonectomy (TPP) and involved field peritonectomy (IFP).

## Discussion

The extent of cytoreduction is reported to have a direct impact on survival in patients with PSM. Optimal cytoreduction is recommended to overcome the prognostic limits imposed by the tumor [18, 19]. CRS has gained a new dimension since the era of peritonectomy procedure, described by Sugarbaker. The utilization of the peritonectomy procedures depends on the tumor spread and extent of invasion in the peritoneal cavity [20]. In advanced ovarian malignancies [21], TPP with en-bloc pelvic resection is reported as a suitable technique which contributes to optimal cytoreduction and thus improved prognosis [22] and such techniques can increase the rate of maximum cytoreduction to 60% [19, 22].

CC score of 1 or less has been associated with improved OS [19]. CC score of 0 was achieved in almost 90% of patients in the present study and this might be the possible reasons for good outcomes with a dedicated team of surgeon, anesthetist, medical oncologist and intensivist. The surgical team has been performing advanced cytoreductions for over 10 years now and is very experienced in the same.

Perioperative IP chemotherapy has been described after achieving optimal CRS in PSM. Cytoreduction score predicts the possibility of benefit from IP therapy and is an independent prognostic factor associated with patient’s survival [19, 23, 24, 25].

HIPEC, a form of perioperative IP therapy is gaining popularity since a decade, is an effective tool for the treatment of microscopic tumor deposits after achieving optimal CRS. The mortality and morbidity rates are reported to vary from 2% to 4% and 30% to 50%, respectively, due to the challenges faced during CRS plus HIPEC [26, 27, 28] suggesting the need for a long learning curve to gain expertise [29]. Our study showed comparable morbidity (42.8% vs. 33.3%) and mortality (4.4% vs. 5.7%) rates in IFP and TPP group, respectively, that stand up well in comparison to those from patients undergoing extensive CRS plus HIPEC.

The basis of TPP comes from the fact that visual inspection of grossly normal looking peritoneum can still harbor tumor deposits. In a prospective study by Johanna et al. [30], it was shown that a microscopically carcinomatous area can have benign appearance on gross inspection in patients with EOC after neoadjuvant chemotherapy. The sensitivity of perioperative visual inspection reached only around 85%, thus questioning the role of IFP especially in NACT or in recurrent setting. In the present study, around 90% patients had some treatment earlier, either in the form of NACT or surgery. In the TPP group, around 30% with normal appearing peritoneum were detected to have microscopic disease after pathological analysis. TPP thus ensures to remove all diseased tissue and limits any marginal miss that can happen from cytoreduction in PSM.

However, the benefits of TPP in patients undergoing HIPEC has been explored only in few studies [3, 9, 10] and it has been underutilized due to concerns of associated morbidity [10]. Retrospective analysis of peritonectomy procedures in patients undergoing HIPEC for mesothelioma by Baratti et al. [9] showed that TPP group had better OS with similar morbidity rates and was recognized as an independent predictor of better prognosis at multivariate analysis. Di Giorgio et al. [3] in a retrospective study of HIPEC in 511 patients with advanced ovarian cancer showed that the completeness of peritonectomy an independent prognostic factor. In the present study, TPP group had decreased overall recurrences when compared to IFP group. The local recurrence rate was 35.7% in TPP group, which was 60% in IFP group which has probably translated to a trend toward better RFS and OS compared with IFP. One important observation was that the benefit of TPP in terms of RFS and OS was obvious after 30 months as seen in from Kaplan–Meier curve. We believe the benefit is mainly due to TPP procedure done over and above CRS plus HIPEC.

This was a retrospective analysis of prospectively collected data. Thus, the inherent exist with this study. However, the baseline parameters were almost comparable between the two groups. The study group was small, but this is one of the largest studies available as per literature and needs longer follow-up to appreciate the oncological outcomes.

## Conclusions

In patients with PSM undergoing HIPEC, TPP can improve the therapeutic efficacy of HIPEC by removing microscopic residual disease. Performing TPP reduces the chance of missing the microscopic disease and therefore minimizes local recurrence as was evident in the study. Improved RFS and OS might be achieved by doing TPP. A prospective randomized multi-institutional study needs to be designed to gain more evidence to define the ideal patient group which will benefit from TPP.
